# Translanguaging pedagogy and learner agency in monolingual Iranian EFL classrooms: An experimental study

**DOI:** 10.1371/journal.pone.0353368

**Published:** 2026-08-14

**Authors:** Morteza Mellati, Sanaz Nademi

**Affiliations:** 1 Shahab Danesh University, Qom, Iran; 2 Islamic Azad University, Tehran, Iran; Bahir Dar University, ETHIOPIA

## Abstract

This study investigated the effects of translanguaging-oriented instruction on Iranian EFL learners’ agency, classroom participation, identity affirmation, and attitudes toward multilingualism. Seventy-eight university students were assigned to an experimental group, which received instruction integrating Persian and English strategically, or a control group, which followed an English-only policy. Data were collected using validated questionnaires at pretest and posttest stages. Analyses included paired- and independent-samples t-tests, ANCOVA, and regression to examine within-group changes, between-group differences, and predictive relationships. Results showed that learners in the experimental group reported significant increases in agency, participation, identity affirmation, and positive attitudes toward multilingualism compared to the control group. Regression analyses indicated that translanguaging-oriented instruction strongly predicted posttest learner agency, even after controlling for pretest scores. The findings suggest that strategic use of learners’ full linguistic repertoire can enhance both cognitive and affective dimensions of EFL learning. The study highlights the potential of translanguaging pedagogy to challenge monolingual ideologies, promote learner autonomy, and foster identity and multilingual awareness in exam-driven EFL contexts. Implications for teaching, curriculum design, and language policy are discussed.

## Introduction

In many EFL (English as a Foreign Language) contexts, classroom instruction continues to be guided by monolingual norms, where the use of learners’ first language is often discouraged or viewed as pedagogically weak. This orientation remains strong in exam-driven systems, where accuracy and target-language exposure are prioritized over meaning-making processes and learner voice. In such contexts, learners are frequently positioned as passive recipients of knowledge, with limited opportunities to draw on their full linguistic resources during classroom interaction [1].

Recent discussions in multilingual education have challenged this monolingual stance by proposing translanguaging as a pedagogical orientation that recognizes learners’ dynamic language practices. Translanguaging views language use not as separated systems, but as a flexible repertoire that learners use to think, communicate, and construct meaning. Research across diverse settings has shown that translanguaging can support comprehension, identity expression, and participation [2–5]. However, much of this work has focused on perceptions, beliefs, or descriptive classroom practices, rather than systematic intervention studies.

In the Iranian EFL context, English classrooms are largely shaped by English-only ideologies, centralized curricula, and high-stakes assessment. Although Persian is the shared linguistic resource of teachers and learners, its classroom use is often unofficial and remains under-theorized. Empirical research that examines how planned translanguaging pedagogy affects learners’ agency-related outcomes in such monolingual-oriented environments is still limited. In particular, there is a lack of experimental evidence that connects translanguaging instruction to learner agency, participation, identity affirmation, and attitudes toward multilingualism [6].

To address this gap, the present study investigates the effects of translanguaging-oriented instruction on Iranian EFL learners in higher education. By adopting an experimental design, the study moves beyond descriptive accounts and examines whether a shift in language policy at the classroom level can lead to measurable changes in learners’ agency and identity-related outcomes. In doing so, the study responds to recent calls in multilingual research for more intervention-based and context-sensitive investigations [7,8].

## Review of the Literature

### Theoretical background: Translanguaging and learner agency

Translanguaging has been conceptualized as both a theory of language and a pedagogical practice that challenges monolingual assumptions in education [9]. From this perspective, language users do not switch between fixed codes, but rather mobilize their linguistic and semiotic resources in fluid ways depending on context and purpose. In educational settings, translanguaging pedagogy allows learners to use their full linguistic repertoire to support learning, interaction, and identity construction.

Learner agency is closely linked to this view of language. Agency refers to learners’ capacity to act, make choices, and position themselves as active participants in learning processes. Studies have suggested that when learners are allowed to use familiar linguistic resources, they are more likely to participate, express opinions, and take ownership of learning tasks [10,11]. From a sociocultural perspective, agency is not a fixed trait, but emerges through interaction and classroom practices. Theoretically, translanguaging pedagogy can create conditions that support learner agency by reducing linguistic anxiety, legitimizing learners’ identities, and reshaping power relations in the classroom [4,12]. However, the extent to which these theoretical claims hold in strongly monolingual EFL contexts requires empirical examination.

### Empirical studies

A growing body of research has explored translanguaging practices across different educational contexts. Qualitative studies have documented how teachers use translanguaging to scaffold learning and negotiate institutional constraints. For example, Abdala [13] and Alharbi and Alqefari [14] reported that teachers often value translanguaging but face tensions between policy expectations and classroom realities. Similar patterns were observed in Jordanian and Saudi EFL contexts, where translanguaging supported meaning-making but also created institutional friction [15,16].

Teacher agency has been a central theme in several studies. Research in Nepal, Japan, and Kazakhstan has shown that teachers actively reshape classroom norms to create translingual spaces, even when official policies promote monolingual instruction [11,17,18]. These studies highlight the role of teachers as mediators of ideology, but they do not directly measure learner outcomes through experimental designs. Learner-focused research has mainly examined perceptions and experiences. Studies in China, Turkey, and the UAE reported that learners view translanguaging positively and associate it with improved understanding and engagement [2,3,19]. Research has also linked translanguaging to identity construction and critical awareness, particularly in marginalized or multilingual settings [20–22].

More recent work has extended translanguaging research to digital and telecollaborative contexts, showing its potential to support interaction and intercultural awareness [23–26]. Despite these advances, most studies rely on qualitative data or mixed-method designs without a clear experimental comparison. Importantly, while some studies have examined motivation and engagement outcomes [27,28], learner agency as a multidimensional construct remains underexplored in experimental EFL research. Furthermore, identity affirmation and attitudes toward multilingualism are often discussed conceptually but are rarely measured as outcome variables in intervention studies.

The reviewed literature reveals several gaps. First, there is a strong dominance of qualitative and perception-based studies, with limited experimental research that tests the effects of translanguaging pedagogy on learner outcomes. Second, most intervention studies have been conducted in multilingual or EMI settings, while monolingual-oriented EFL contexts such as Iran remain underrepresented. Third, learner agency, identity affirmation, and attitudes toward multilingualism are often treated as secondary themes rather than central outcome variables [29]. Moreover, previous research has largely focused on teacher beliefs and practices, leaving learners’ agency-related responses to pedagogical translanguaging less examined. There is also limited evidence on whether translanguaging can challenge monolingual ideologies at the classroom level in exam-oriented systems. To address these gaps, the present study adopts an experimental design to examine the impact of translanguaging-oriented instruction on Iranian EFL learners’ agency, classroom participation, identity affirmation, and attitudes toward multilingualism. By doing so, the study contributes empirical evidence to ongoing debates on multilingual pedagogy and provides context-specific insights for EFL education in monolingual policy environments.

### Research question

To what extent does translanguaging-oriented instruction influence Iranian EFL learners’ agency, classroom participation, identity affirmation, and attitudes toward multilingualism in monolingual classroom settings?

## Materials & Methods

### Participants

The participants of this study were 78 Iranian EFL learners enrolled in general English courses at a public university in Iran. The participants were selected through convenience sampling because the researchers had institutional access to the target classes during the data collection period. All participants were native speakers of Persian and had learned English as a foreign language through formal educational instruction. The sample included both male and female undergraduate students between 18 and 24 years of age who were majoring in non-English-related academic fields. English was a compulsory component of their university curriculum. Based on institutional placement procedures, the learners were classified at lower-intermediate to intermediate proficiency levels. None of the participants had lived in an English-speaking country or reported extensive exposure to English beyond classroom settings. The participants were drawn from two intact classes. One class (n = 39) was assigned as the experimental group and received translanguaging-oriented instruction, whereas the other class (n = 39) served as the control group and followed an English-only instructional approach. Because the classes had already been formed administratively before the beginning of the semester, random assignment at the individual level was not feasible.

Ethical approval for the study was obtained from the Research Ethics Committee of the Faculty of Humanities at the host public university prior to data collection. The study procedures complied with institutional ethical guidelines for research involving human participants. Before the beginning of the study, the learners received an explanation regarding the aims of the research, the nature of participation, classroom procedures, and the intended use of the collected data. Written informed consent was obtained from all participants before the administration of the pretest instruments. Participation was fully voluntary, and the learners were informed that they could withdraw from the study at any stage without academic penalty or negative consequences. To protect participants’ privacy, questionnaire responses were anonymized during the coding process, and no personally identifying information was included in the dataset or reporting procedures. The participants were also assured that their responses would be used exclusively for research purposes and would not influence course grades, instructor evaluations, or academic standing. Because all participants were adults at the time of data collection, parental consent was not required.

### Instruments

Data were collected using four self-report questionnaires that measured learner agency, classroom participation, identity affirmation, and attitudes toward multilingualism. All instruments were administered at the pretest and posttest stages. The questionnaires were translated into Persian using a forward–backward translation procedure. Two university instructors in applied linguistics reviewed the translated versions to confirm content clarity and contextual appropriateness. A pilot study with a similar group of learners was conducted to check item clarity and reliability before the main data collection.

#### Learner agency questionnaire.

Learner agency was assessed using an adapted questionnaire developed for educational and language learning settings [30,31]. The instrument consisted of 12 items designed to capture learners’ perceived control over learning activities, willingness to express opinions, initiative in classroom participation, and sense of responsibility toward language learning. The items were reviewed by three specialists in applied linguistics and educational psychology to evaluate clarity, contextual appropriateness, and conceptual alignment with learner agency in Iranian EFL classrooms. Minor wording revisions were made to improve comprehensibility and reduce possible ambiguity for participants. Because learner agency has been conceptualized as a multidimensional and context-sensitive construct, the factor structure of the adapted scale was examined within the present sample rather than relying solely on evidence reported in earlier studies. A confirmatory factor analysis (CFA) was conducted using AMOS 24 on the pretest data. The results supported a three-factor structure representing perceived control, learner voice, and active engagement. Model fit indices indicated an acceptable fit to the data (χ^2^/df = 2.11, CFI = .94, TLI = .92, RMSEA = .058). All factor loadings exceeded.60 and were statistically significant. Although the scale reflected multiple interrelated dimensions conceptually, the correlations among the three factors were relatively high; therefore, an overall learner agency score was calculated and used in the main statistical analyses. At the same time, the multidimensional structure was retained theoretically to acknowledge the complex and socially situated nature of agency. The use of a self-report Likert-scale instrument was considered appropriate for the purposes of the present study because the research focused on learners’ perceived sense of agency within classroom participation and language learning processes. Previous sociocultural research has suggested that agency is not only enacted through interaction but also mediated by learners’ interpretations of their positioning, voice, and opportunities for participation [30]. In this sense, learners’ self-perceptions provide meaningful access to how they experience and negotiate agency in classroom contexts, even though agency may also emerge dynamically through interactional practices. The questionnaire was therefore used to capture participants’ subjective orientations toward classroom involvement rather than to claim direct measurement of interactional behavior itself. Internal consistency reliability was examined using Cronbach’s alpha, which demonstrated acceptable reliability for the overall scale at both measurement points.

#### Classroom participation questionnaire.

Classroom participation was assessed using a questionnaire adapted from established participation scales in EFL research [32,33]. The instrument consisted of 10 items measuring learners’ verbal contribution, involvement in group activities, and willingness to engage in classroom tasks. Content validity was established through adaptation from previous validated instruments and expert judgment to ensure relevance to EFL classroom interaction. Previous studies have reported acceptable construct validity for similar participation measures, showing clear associations with engagement and classroom behavior. In the current study, reliability analysis showed satisfactory internal consistency, indicating that the items consistently measured classroom participation among Iranian EFL learners.

#### Identity affirmation scale.

Learners’ identity affirmation was measured using an adapted scale that examined learners’ sense of legitimacy, confidence, and comfort in using English as multilingual speakers [34]. The scale included 8 items derived from previous identity and investment research in second language learning. Content validity was supported through alignment with theoretical discussions of language learner identity and expert review. Construct validity of similar identity-related instruments has been reported in prior studies through factor analytic evidence. In the present study, reliability was examined using Cronbach’s alpha, and the results indicated acceptable internal consistency for the scale, suggesting that the items functioned coherently in the Iranian EFL context.

### Attitudes toward multilingualism scale

Attitudes toward multilingualism were measured using a 10-item questionnaire adapted from established instruments in multilingual education research [35]. The scale examined learners’ beliefs about the value of using multiple languages in learning and communication. Content validity was ensured by grounding the items in multilingualism theory and by expert evaluation. Previous research has shown that similar scales demonstrate acceptable construct validity across different educational contexts. In the current study, internal consistency reliability was calculated, and the results showed acceptable reliability coefficients, indicating that the scale reliably measured learners’ attitudes toward multilingualism. All of the instruments demonstrated acceptable levels of validity and reliability and were considered appropriate for investigating learner agency, participation, identity affirmation, and attitudes toward multilingualism in Iranian EFL classrooms.

### Procedure

The study was conducted over one academic term using a quasi-experimental pretest–posttest control group design. Prior to data collection, permission to conduct the study was obtained from the university administration and course instructors. Participants were informed about the aims of the study, the voluntary nature of participation, and the confidentiality of their responses. Written consent was obtained from all learners before the administration of the pretest instruments. During the first week of the semester, both groups completed questionnaires measuring learner agency, classroom participation, identity affirmation, and attitudes toward multilingualism under the supervision of the researchers. Following the pretest phase, the instructional intervention was implemented for six consecutive weeks. Both groups studied the same textbook units, followed the same assessment schedule, and completed comparable language-learning tasks. The only systematic difference between the groups concerned the instructional language policy and classroom interaction procedures.

#### Instructional treatment details.

The experimental group received translanguaging-oriented instruction designed around planned and pedagogically structured use of Persian alongside English. Translanguaging was not treated as unrestricted alternation between languages; rather, it was incorporated into predetermined stages of classroom activities for specific learning purposes. Across the intervention period, approximately 20–30% of classroom interaction involved Persian use, while English remained the principal medium of instruction and output production. Persian was primarily used during meaning negotiation, collaborative planning, metalinguistic reflection, and clarification of complex task instructions. Final oral and written products were generally completed in English. Each instructional session lasted approximately 90 minutes and followed a relatively stable sequence. The opening phase (10–15 minutes) was conducted mainly in English and included lesson introduction, vocabulary activation, and short warm-up activities. During the collaborative task phase (25–35 minutes), learners in the experimental group were allowed to use Persian strategically while discussing ideas, negotiating meanings, comparing grammatical structures, or preparing responses before presenting them in English. This stage represented the main translanguaging space within the lesson. The production phase (20–25 minutes) required learners to complete oral presentations, role-plays, paragraph writing, or group reporting primarily in English. The final reflective phase (10–15 minutes) occasionally included brief Persian use to help learners discuss learning difficulties, evaluate task strategies, or connect classroom content to personal experiences.

The translanguaging practices were implemented through several predefined instructional strategies. First, learners were encouraged to brainstorm ideas in Persian before reorganizing them into English responses. Second, peer discussions allowed bilingual negotiation when learners encountered lexical or conceptual difficulties. Third, contrastive explanation tasks required students to compare selected English expressions with Persian equivalents to increase awareness of pragmatic or semantic differences. Fourth, reflective discussion activities invited learners to express emotional reactions, classroom concerns, or identity-related experiences using either language before summarizing key points in English. Several recurring classroom tasks required planned translanguaging use. In one activity, learners discussed culturally loaded English expressions in Persian small-group discussions and then collaboratively prepared English explanations for class presentation. In another task, students analyzed short reading passages in English, discussed the meanings of unfamiliar expressions in Persian, and subsequently produced English summaries. A third activity involved reflective journal discussions in which learners first exchanged ideas bilingually in pairs and later submitted brief English reflections about classroom participation and language learning experiences.

The instructional design allowed limited spontaneous translanguaging when communicative breakdowns occurred or when learners needed clarification during collaborative interaction. However, such use remained pedagogically bounded rather than unrestricted. The instructor redirected learners toward English once comprehension had been established or task planning had been completed. Unplanned Persian use was therefore not treated as inappropriate behavior, but it was moderated to maintain the instructional focus on English language development. In the control group, instruction followed an English-only policy. Classroom explanations, peer interaction, task completion, and feedback procedures were conducted in English. Learners were regularly reminded to avoid Persian during classroom interaction, and the instructor monitored group activities to maintain consistency with the instructional condition. Occasional isolated Persian expressions naturally occurred, particularly during moments of confusion; however, these instances were immediately redirected toward English and were not incorporated into instructional procedures.

To minimize teacher-related variability, the same instructor taught both classes using lesson plans jointly prepared with the researchers. Before the intervention, the instructor participated in an orientation session focused on translanguaging principles, treatment procedures, classroom timing, and activity sequencing. To examine implementation fidelity, the researchers conducted periodic classroom observations using a structured observation checklist that documented instructional language choice, duration of Persian use, adherence to lesson stages, and the nature of classroom interaction. Observation records indicated that the intervention procedures were implemented consistently across the six-week period. At the end of the intervention, posttest questionnaires were administered under conditions similar to those of the pretest phase. Participants were reminded that their responses would remain confidential and would not influence their academic grades. After data collection, questionnaire responses were coded and entered into SPSS version 27 for statistical analysis. Cases with incomplete responses were screened prior to analysis, and all remaining datasets were retained for the final analyses.

#### Data analysis.

The collected data were analyzed using SPSS (version 27). Prior to the main analyses, the data were screened for missing values, outliers, and entry errors. Cases with substantial missing data were excluded, while minor missing values were handled using mean substitution. The assumptions of normality, linearity, and homogeneity of variance were examined through descriptive statistics, skewness and kurtosis values, and Levene’s test. The results indicated that the data met the assumptions required for parametric analyses. Descriptive statistics, including means and standard deviations, were calculated for all variables at the pretest and posttest stages. Internal consistency reliability for each questionnaire was assessed using Cronbach’s alpha coefficients. Correlation analyses were conducted to examine the relationships among learner agency, classroom participation, identity affirmation, and attitudes toward multilingualism.

To examine within-group changes over time, paired-samples t-tests were conducted for each group separately. Independent-samples t-tests were then used to compare posttest scores between the experimental and control groups. To control for possible pretest differences, analysis of covariance (ANCOVA) was conducted with posttest scores as the dependent variables and pretest scores as covariates. In addition, multiple regression analysis was performed to examine whether translanguaging-oriented instruction predicted learner agency at the posttest stage. Group membership was entered as a predictor, while pretest agency scores were controlled. Effect sizes were calculated using Cohen’s d and partial eta squared to estimate the magnitude of the observed effects. The level of statistical significance was set at p < .05 for all analyses.

## Results

### Preliminary analyses

Prior to conducting the main analyses, the data were screened for accuracy and statistical assumptions. No extreme outliers were detected. Skewness and kurtosis values for all variables ranged between −1.12 and +1.05, indicating acceptable univariate normality. Levene’s test results were non-significant for all posttest variables (p > .05), suggesting homogeneity of variance. Cronbach’s alpha coefficients confirmed acceptable internal consistency for all scales.

As shown in Table 1, both groups demonstrated similar mean scores at the pretest stage across all variables, suggesting initial comparability. At the posttest stage, the experimental group reported noticeably higher mean scores on learner agency, classroom participation, identity affirmation, and attitudes toward multilingualism. Reliability indices ranged from.78 to.87, indicating acceptable internal consistency across measurement points.

**Table 1:** Descriptive statistics and reliability coefficients for study variables.

Variable	Time	Group	Mean	SD	Cronbach’s α
**Learner Agency**	Pre	Experimental	3.13	0.48	.83
		Control	3.11	0.46	.84
	Post	Experimental	3.84	0.52	.83
		Control	3.24	0.42	.81
**Classroom Participation**	Pre	Experimental	2.92	0.54	.71
		Control	2.92	0.56	.74
	Post	Experimental	3.72	0.59	.88
		Control	3.04	0.45	.83
**Identity Affirmation**	Pre	Experimental	3.08	0.41	.82
		Control	3.02	0.49	.84
	Post	Experimental	3.85	0.55	.87
		Control	3.16	0.44	.84
**Attitudes toward Multilingualism**	Pre	Experimental	3.24	0.44	.85
		Control	3.12	0.54	.84
	Post	Experimental	4.05	0.51	.84
		Control	3.24	0.55	.81

The paired-samples t-test results in Table 2 indicate that learners in the experimental group experienced significant increases across all four variables after the intervention. In contrast, the control group did not show statistically significant changes from pretest to posttest. These findings suggest that the observed improvements were associated with the instructional approach rather than normal classroom progression.

**Table 2:** Paired-samples T-tests for pretest and posttest scores.

Variable	Group	t	df	p
**Learner Agency**	Experimental	8.45	38	<.001
	Control	1.37	38	.181
**Classroom Participation**	Experimental	7.91	38	<.001
	Control	1.28	38	.244
**Identity Affirmation**	Experimental	7.25	38	<.001
	Control	1.09	38	.303
**Attitudes toward Multilingualism**	Experimental	8.61	38	<.001
	Control	1.55	38	.136

As reported in Table 3, the experimental group significantly outperformed the control group on all posttest measures. The effect sizes were large across all variables, indicating that translanguaging-oriented instruction had a strong practical impact on learners’ agency-related and identity-related outcomes.

**Table 3:** Independent-samples T-tests comparing posttest scores.

Variable	t	df	p	Cohen’s d
**Learner Agency**	5.78	76	<.001	1.31
**Classroom Participation**	5.62	76	<.001	1.27
**Identity Affirmation**	5.11	76	<.001	1.16
**Attitudes toward Multilingualism**	6.04	76	<.001	1.37

After controlling for pretest scores, ANCOVA results in Table 4 revealed a significant main effect of instructional condition on all dependent variables. Partial eta squared values ranged from.25 to.31, suggesting moderate to large effects. These results confirm that the posttest differences between groups cannot be attributed to initial score variation.

**Table 4:** ANCOVA results for posttest scores (controlling for pretest).

Dependent Variable	F	p	Partial η^2^
**Learner Agency**	31.24	<.001	.29
**Classroom Participation**	28.67	<.001	.27
**Identity Affirmation**	25.11	<.001	.25
**Attitudes toward Multilingualism**	33.89	<.001	.31

The regression model explained 47% of the variance in learner agency at the posttest stage. Instructional group emerged as a strong predictor of learner agency, even after controlling for pretest scores. The findings in Table 5 indicate that participation in translanguaging-oriented instruction substantially contributed to learners’ perceived agency in the classroom. The results of the study provide consistent evidence that translanguaging-oriented pedagogy positively influenced learner agency, participation, identity affirmation, and attitudes toward multilingualism. The pattern of findings across multiple statistical tests supports the effectiveness of allowing strategic use of learners’ full linguistic repertoires in a context traditionally guided by monolingual norms.

**Table 5:** Multiple regression predicting learner agency (posttest).

Predictor	B	SE B	β	t	p
**Pretest Agency**	0.41	0.09	.38	4.56	<.001
**Instructional Group**	0.62	0.11	.51	5.74	<.001

R^2^ =.47, Adjusted R^2^ =.45, F(2, 75) = 33.21, p <.001.

## Discussion

The present study examined the effects of translanguaging-oriented instruction on Iranian EFL learners’ agency, classroom participation, identity affirmation, and attitudes toward multilingualism. The findings showed that learners who experienced planned and guided use of Persian alongside English reported noticeable gains across all outcome variables, whereas those in the English-only group showed limited or marginal change. This pattern suggests that allowing learners to draw on their full linguistic repertoire can support their engagement and sense of involvement in classroom activities. Rather than hindering English learning, the strategic inclusion of Persian appeared to facilitate meaning-making processes and reduce communicative barriers. In this way, translanguaging-oriented instruction functioned as a pedagogical resource that supported both cognitive engagement, such as understanding tasks and participating in discussions, and affective dimensions, such as confidence, sense of belonging, and willingness to contribute.

The results are consistent with previous studies indicating that translanguaging practices enhance comprehension, participation, and identity construction in EFL and multilingual classrooms [2–4]. Research conducted in Jordan, Saudi Arabia, and China has shown that strategic L1 use may help teachers scaffold cognitively demanding content while also encouraging learners to participate more actively in classroom interaction [15,27]. Parallel findings have been reported in Japan, Nepal, and Turkey, where translanguaging practices appeared to create interactional space for meaning negotiation, collaborative interpretation, and shared understanding, even in institutional contexts that formally promoted English-only policies [11,18,21].

The present study extends this line of research by providing experimental evidence that translanguaging-oriented instruction can contribute not only to participation and interactional engagement but also to learners’ perceptions of agency and identity affirmation in monolingual EFL classrooms. Unlike many earlier investigations that relied mainly on interview data, classroom observations, or self-reported perceptions without comparison groups, the current study compared two instructional conditions over a structured intervention period and identified measurable differences across several learner-related outcomes.

The findings may also be interpreted within a broader affective-pedagogical framework. Recent research in Iranian EFL settings has increasingly emphasized the relationship between classroom task design and learners’ psychological experiences. For instance, Mirzaei et al. [23] reported that picture-based storytelling, description-based drawing, and song-based drawing tasks significantly influenced learners’ self-esteem and psychological well-being. Their findings suggest that pedagogical practices which provide learners with opportunities for creativity, emotional expression, and collaborative engagement can positively shape affective dimensions of language learning. Although the focus of the present study was translanguaging-oriented instruction rather than task-based emotional engagement itself, there appears to be an important conceptual overlap between these approaches. In both cases, learners are given greater discursive space to express meaning, negotiate understanding, and participate without excessive fear of linguistic inadequacy.

From this perspective, translanguaging may function not only as a linguistic scaffold but also as an affective resource that reduces interactional pressure and strengthens learners’ sense of legitimacy within the classroom community. The increase observed in learner agency and identity affirmation in the experimental group arguably reflects this broader psychological dimension. When learners are permitted to draw selectively on their existing linguistic repertoires, classroom participation may become less restrictive and more personally meaningful, particularly in contexts where English-only ideologies have traditionally positioned the first language as an obstacle rather than a pedagogical resource. Such conditions may contribute indirectly to learners’ confidence, self-expression, and emotional comfort during classroom interaction.

The convergence between the present findings and those reported by Mirzaei et al. [23] may therefore indicate that learner-centered and linguistically flexible pedagogies share an underlying affective mechanism. Classroom environments that recognize learners’ linguistic and emotional resources appear more likely to support active engagement and positive self-perceptions. This interpretation remains tentative because psychological well-being and self-esteem were not measured directly in the current study. Nevertheless, the findings seem to support the growing argument that instructional approaches in EFL classrooms should be evaluated not only in terms of linguistic achievement but also in relation to learners’ emotional and identity-related experiences.

The study contributes to the growing body of translanguaging research by showing that translanguaging is not limited to facilitating comprehension but also plays a central role in shaping learners’ sense of agency in exam-driven and monolingual-oriented EFL contexts. The results indicate that translanguaging-oriented instruction served as a strong predictor of learner agency and also supported learners’ identity affirmation and openness toward multilingualism. When learners were allowed to use Persian as a legitimate resource, they appeared more willing to take initiative, express opinions, and participate in classroom interaction. This suggests that classroom policies that acknowledge learners’ L1 may enhance engagement and self-confidence rather than reduce exposure to English. In addition, the findings support sociocultural views of agency by showing that learner agency is not a fixed personal trait but develops through interaction and instructional practices [9,12]. By empirically linking translanguaging pedagogy to agency development, the study offers further support for rethinking rigid monolingual policies in EFL education.

### Implications

From a theoretical perspective, the findings of the present study support the view that learner agency develops through social interaction and is mediated by the language resources available in the classroom. When learners were encouraged to use both Persian and English, they were able to participate more actively in meaning-making processes, take initiative in classroom tasks, and exercise choice in how they expressed ideas. Translanguaging pedagogy thus functioned as a mediating tool that connected learners’ linguistic resources with their capacity to act and engage. The observed growth in identity affirmation further supports the argument that translanguaging allows learners to draw on personal and cultural experiences when using language, rather than separating language learning from identity construction. In this sense, the findings challenge monolingual ideologies that position L1 use as interference and instead confirm that recognizing learners’ full linguistic repertoires can contribute to agency development and identity negotiation in EFL classrooms [4,20].

From a practical perspective, the results suggest several implications for EFL teaching and curriculum planning. Teachers may enhance learner engagement and classroom participation by adopting translanguaging strategies, such as allowing bilingual peer discussion, providing explanations or feedback in learners’ L1 when needed, and designing reflective activities that encourage learners to move between languages. These practices can reduce communicative pressure and support learners who may hesitate to participate under English-only conditions. At the institutional level, curriculum designers and policymakers may consider adopting more flexible language policies that permit strategic L1 use, rather than enforcing strict English-only approaches. Such flexibility may help create learning environments that are more inclusive and supportive, particularly in exam-oriented contexts where learners often experience anxiety and limited opportunities for meaningful interaction. By legitimizing translanguaging practices, educational systems can better align classroom instruction with learners’ linguistic realities and promote sustained engagement in EFL learning.

### Limitations

Despite the contributions of the present study, several limitations should be acknowledged. First, the use of intact classes restricted full random assignment of participants to the experimental and control conditions. Although pre-test equivalence was established, this design choice may have allowed unmeasured classroom-level factors, such as peer dynamics or prior instructional experiences, to influence the outcomes. As a result, causal interpretations of the effects of translanguaging-oriented instruction should be made with caution. Second, the study was conducted at a single Iranian university, with participants drawn from a relatively homogeneous academic context. This setting may limit the transferability of the findings to other EFL contexts, educational levels, or sociolinguistic environments where language policies and learner backgrounds differ.

Third, the instructional intervention lasted six weeks, which may not be sufficient to capture the sustained or delayed effects of translanguaging on learner agency, identity affirmation, and attitudes toward multilingualism. Changes in these constructs may evolve over longer periods as learners gain continued exposure to flexible language practices. Finally, the study relied primarily on self-report questionnaires to measure learner perceptions and attitudes. While these instruments provided valuable insights into learners’ experiences, they may have been subject to social desirability bias, particularly in contexts where learners are aware of the pedagogical goals of the intervention. This reliance may have influenced how participants reported their engagement and views toward multilingual practices.

### Suggestion for future studies

Future research may address the above limitations by adopting more robust and diverse research designs. Multi-site studies involving different universities, regions, or educational levels could help examine whether the observed effects of translanguaging-oriented instruction hold across varied EFL contexts. Longitudinal designs would also be valuable in tracing how learner agency, identity affirmation, and attitudes toward multilingualism develop over extended periods of instructional exposure. In addition, future studies may incorporate multiple data sources, such as classroom observations, teacher reflective journals, and learner interviews, to triangulate quantitative findings and provide deeper insights into classroom processes.

Further research could also examine the role of translanguaging across different proficiency levels and assessment settings, particularly in high-stakes exam contexts where English-only norms are strongly enforced. Such work may clarify how instructional language policies interact with learner agency and participation. Moreover, extending translanguaging research to technology-mediated and online EFL environments would be timely. Investigating digital platforms and virtual classrooms may build on existing work by Sun [24] and Turnbull et al. [26], and help determine how translanguaging practices function in online interaction, collaborative tasks, and digitally supported language learning.

## Conclusion

In conclusion, the present study offers empirical support for the effectiveness of translanguaging-oriented instruction in enhancing Iranian EFL learners’ agency, classroom participation, identity affirmation, and attitudes toward multilingualism within a predominantly monolingual instructional context. The experimental findings indicate that when learners are permitted to draw on both Persian and English in a systematic and pedagogically guided manner, they become more willing to participate, take initiative in classroom interaction, and position themselves as legitimate language users. These outcomes suggest that learner engagement is not solely a function of linguistic proficiency, but is closely linked to how classroom language policies shape learners’ sense of voice and ownership in the learning process.

More broadly, the study demonstrates that translanguaging pedagogy can serve as a practical response to the constraints of English-only norms that often characterize exam-oriented EFL settings. By legitimizing learners’ full linguistic repertoires, teachers can create learning environments that are more inclusive and responsive to learners’ identities and sociocultural backgrounds. Such environments appear to support not only cognitive engagement, but also affective dimensions of learning, including confidence, belonging, and positive orientations toward multilingualism. Taken together, the findings underscore the transformative potential of translanguaging as both a pedagogical approach and a theoretical lens, contributing to ongoing discussions in multilingual education and offering direction for future research and practice in global EFL contexts.

## Supporting information

S1 Data Raw data. (XLSX)

## References

[1] Baazaoui H, Cheniti-Belcadhi L, Laymani D, Guyeux C. Bibliometric review of AI-driven code-switching in multilingual education. J Comput Educ. 2025. doi: 10.1007/s40692-025-00378-7

[2] Jiang ZW, Zhang LJ, Mohamed N. Researching translanguaging as a feasible pedagogical practice: evidence from chinese english-as-a-foreign-language students’ perceptions. RELC Journal. 2022;53(2):371–90. doi: 10.1177/00336882221113653

[3] Liu D, Deng Y, Wimpenny K. Students’ perceptions and experiences of translanguaging pedagogy in teaching English for academic purposes in China. Teach High Educ. 2022;29(5):1234–52. doi: 10.1080/13562517.2022.2129961

[4] Sah PK, Li G. Toward linguistic justice and inclusion for multilingual learners: implications of selective translanguaging in English-medium instruction classrooms. Learn Instruct. 2024;92:101904. doi: 10.1016/j.learninstruc.2024.101904

[5] Yüzlü MY, Dikilitaş K. EFL teachers learning to translanguage through loop input: implications for their identity-reconstruction. Lang Aware. 2024;34(2):231–56. doi: 10.1080/09658416.2024.2429657

[6] Ghajarieh A, Mozaheb MA, Ghaziyani ZA. Playing with words across visual humor in an Iranian EFL context with Arab students: pedagogical translanguaging for enhancement of multicultural spaces in language education. Int J Educ Res. 2024;124:102278. doi: 10.1016/j.ijer.2023.102278

[7] Uras Eren E. Integrating translanguaging pedagogy in EFL higher education: student perceptions and implications for teacher education in monolingual contexts. Learn Instruct. 2026;102:102297. doi: 10.1016/j.learninstruc.2025.102297

[8] Wang F, Ai B. Towards necessities and challenges of implementing translanguaging pedagogy in secondary EFL education in China. Language Teaching Research. 2024. doi: 10.1177/13621688241306708

[9] Liu Y, Fang F. Translanguaging theory and practice: how stakeholders perceive translanguaging as a practical theory of language. RELC J. 2020;53(2):391–9. doi: 10.1177/0033688220939222

[10] Malgoubri I. Empowering teachers and reclaiming agency through culturally sustaining arts-based pedagogy in English language teaching. Teaching and Teacher Education. 2026;171:105328. doi: 10.1016/j.tate.2025.105328

[11] Phyak P, Sah PK, Ghimire NB, Lama A. Teacher agency in creating a translingual space in Nepal’s multilingual english-medium schools. RELC J. 2022;53(2):431–51. doi: 10.1177/00336882221113950

[12] Slaughter Y, Cross R. Challenging the monolingual mindset: Understanding plurilingual pedagogies in English as an Additional Language (EAL) classrooms. Lang Teach Res. 2020;25(1):39–60. doi: 10.1177/1362168820938819

[13] Abdala J. Reimagining language use in classrooms: pre-service teachers’ beliefs and practices of translanguaging pedagogy in Tanzanian secondary education. Int J Multilingual. 2025;:1–27. doi: 10.1080/14790718.2025.2601275

[14] Alharbi MAS, Alqefari AN. Innovative translanguaging strategies in Saudi Arabia: a qualitative analysis of EFL Saudi teachers. Innov Lang Learn Teach. 2025;:1–21. doi: 10.1080/17501229.2025.2552868

[15] Almashour M, Aldamen H, Jarrah M, Al-Deaibes M. Translanguaging in Jordanian EFL assessment: cognitive scaffolding, identity expression, and institutional friction. System. 2026;137:103917. doi: 10.1016/j.system.2025.103917

[16] Rajendram S, Burton J, Wong W. Online translanguaging and multiliteracies strategies to support K‐12 multilingual learners: Identity texts, linguistic landscapes, and photovoice. TESOL J. 2022;13(4). doi: 10.1002/tesj.685

[17] Agbo SA, Kenshinbay T, Kazhiakbarova R, Nebessayeva Z, Myrzabek A. Collaboration, empowerment, education, and action: Kazakhstan’s teachers’ creative maladjustment in utilizing translanguaging as a pedagogical strategy. Int J Educ Reform. 2025. doi: 10.1177/10567879251400325

[18] Seki K. Negotiating monolingual norms: teacher agency and translanguaging pedagogy in an English-immersion school in Japan. Lang Learn J. 2025:1–14. doi: 10.1080/09571736.2025.2581070

[19] Kinuthia H. The recontextualisation and cultural compatibility of student-centred education: the case of the United Arab Emirates. High Educ. 2023;87(4):1009–26. doi: 10.1007/s10734-023-01049-1

[20] Kayi‐Aydar H, Green‐Eneix C. Shared identities through translanguaging practices in the multilingual mariachi classroom. TESOL J. 2019;10(4). doi: 10.1002/tesj.502

[21] Toker Ş, Olğun Baytaş M. Grappling with the transformative potential of translanguaging pedagogy in an elementary school with Syrian refugees in post-coup Turkey. Int Multilingual Res J. 2021;16(2):148–62. doi: 10.1080/19313152.2021.2004768

[22] Thongwichit N, Ulla MB, Parba J. Translanguaging for social justice in Thailand’s language classrooms: a classroom ethnography. Int J Multilingual. 2025;23(1):46–62. doi: 10.1080/14790718.2025.2517334

[23] Mirzaei F, Ahangaran F, Pouyan A. Examining the roles of picture-based storytelling, description-based drawing, and song-based-drawing tasks in EFL learners’ self-esteem and psychological well-being. Int J Pract Pedagog Issues English Educ. 2025;3(4):1–22. doi: 10.22034/ijpie.2025.522291.1103

[24] Sun L. Translanguaging pedagogy on the digital stage: exploring Chinese undergraduates’ English grammar learning through DingTalk platform. Humanit Soc Sci Commun. 2024;11(1). doi: 10.1057/s41599-024-03771-2

[25] Yeh H-C, Qi GY, Lin M-W, Chen N-S. Oral translanguaging in telecollaboration: Effects on EFL learner intercultural awareness, learning and communicative competence. System. 2024;124:103357. doi: 10.1016/j.system.2024.103357

[26] Turnbull J, Yazan B, Uzum B, Akayoglu S. ‘That was crazy’: Confronting monolingual ideologies and courting translanguaging in international telecollaboration. Lang Teach Res. 2025. doi: 10.1177/13621688251349519

[27] Tai KWH. Transitioning from L1 medium of iInstruction to L2 English medium instruction: the role of translanguaging in contributing to EFL students’ language learning motivation. Int J App Linguistics. 2025. doi: 10.1111/ijal.12820

[28] Turnbull B. Enhancing motivation and listening comprehension among Japanese EFL students through translanguaging practices. Int J Appl Linguistics. 2026. doi: 10.1111/ijal.70072

[29] Ibna Seraj PM, Afrin M, Klimova B. Exploring the key research features and themes on the issue of translanguaging and EFL learners’ writing skills. Soc Sci Humanit Open. 2026;13:102401. doi: 10.1016/j.ssaho.2025.102401

[30] Mercer S. The complexity of learner agency. Appl Linguistics. 2012;33(1):41–59.

[31] Oga-Baldwin WLQ, Nakata Y. Engagement, gender, and motivation: a predictive model for Japanese young language learners. System. 2017;65:151–63. doi: 10.1016/j.system.2017.01.011

[32] Dörnyei Z, Kormos J. The role of individual and social variables in oral task performance. Lang Teach Res. 2000;4(3):275–300. doi: 10.1177/136216880000400305

[33] Peng J-E. Towards an ecological understanding of willingness to communicate in EFL classrooms in China. System. 2012;40(2):203–13. doi: 10.1016/j.system.2012.02.002

[34] Gao Y, Jia Z, Zhou Y. EFL learning and identity development: a longitudinal study in 5 universities in China. J Lang Identity Educ. 2015;14(3):137–58. doi: 10.1080/15348458.2015.1041338

[35] Lasagabaster D. Language attitudes in multilingual contexts. Lang Aware. 2017;26(1):1–7. doi: 10.1111/modl.12414

